# Using the SPE and Micro-HPLC-MS/MS Method for the Analysis of Betalains in Rat Plasma after Red Beet Administration

**DOI:** 10.3390/molecules22122137

**Published:** 2017-12-04

**Authors:** Tomasz Sawicki, Jerzy Juśkiewicz, Wiesław Wiczkowski

**Affiliations:** Institute of Animal Reproduction and Food Research, Polish Academy of Sciences in Olsztyn, Tuwima 10, 10-748 Olsztyn, Poland; t.sawicki@pan.olsztyn.pl (T.S.); j.juskiewicz@pan.olsztyn.pl (J.J.)

**Keywords:** betalains, plasma, solid phase extraction (SPE), micro-HPLC-MS/MS, validation method

## Abstract

The objective of this study was to develop a simple and reproducible method for the qualitative and quantitative analysis of betalains in plasma samples, based on Solid Phase Extraction (SPE) and micro-high performance liquid chromatography coupled with mass spectrometry (micro-HPLC-MS/MS). The eight betalain compounds detected and quantified were characterized in the fortified rat blood plasma samples. The developed method showed a good coefficient of determination (R^2^ = 0.999), good recovery, precision, and appropriate limits of detection (LOD) and quantification (LOQ) for these compounds. Application of this method for the treatment of rat plasma samples collected after the betalain preparation administration, for the first time, revealed the presence of native betalains and their metabolites in plasma samples. Moreover, among them, betanin (2.14 ± 0.06 µmol/L) and isobetanin (3.28 ± 0.04 µmol/L) were found at the highest concentration. The results indicated that the combination of an SPE method with a micro-HPLC-MS/MS analysis may be successfully applied for the determination of betalains in the blood plasma.

## 1. Introduction

Betalains are water-soluble natural pigments that may be divided into two groups, red-violet betacyanins and yellow-orange betaxanthins [1,2]. These compounds have a number of health-promoting properties, exhibiting strong antioxidant, antiviral, anticancer, antilipidemic and antibacterial activity [3,4]. Betalains are found in plants of *Caryophyllales* order, among others Swiss chard (*Beta vulgaris* L. ssp. *cicla*) [5], cactus pear (*Opuntia ficus-indica*) [6], pitaya (*Hylocereus polyrhizus*) [7], ulluco (*Ullucus tuberosus*) [8], amaranth (*Amaranthus* sp.) [3], and red beetroot (*Beta vulgaris* L. ssp. *vulgaris*) [9]. Among the above-mentioned plants, red beetroot constitutes the richest source of these compounds. Despite a limited prevalence in the plant kingdom, betalains are widely used in the food industry [10,11]. These natural compounds are successfully used for the coloring of such food products as ice cream, jam, yoghurt, marmalade, and sweets. In a dried form, they are frequently added to many types of tea. Moreover, application of betalains as food pigments is approved by the European Union, with this group of substances being labeled as E-162 [12].

Despite the abundance of betalains in plant-derived food products and an increasing number of articles related to the biological properties of betalains [3,4], their profile in plasma has not been well-recognized yet. Taking these facts into account, it is necessary to track the fate of these substances in the human body after ingestion. This requires a specific treatment of body fluids such as blood plasma, which would reduce the loss of these compounds, as well as an adequately sensitive and accurate method of analysis. To our knowledge, there have only been three studies investigating the concentration of betalains in the samples of plasma. The first study in plasma samples showed that only one compound from the group of betaxanthins (indicaxanthin) originated from cactus pear [13]. Unfortunately, this paper does not provide any methods of preparing plasma samples for analysis. The second study by Tesoriere et al. [14] involved a method based on the extraction of plasma samples with a chloroform/methanol mixture and the analysis with HPLC-DAD. In the samples analyzed, only two native compounds from the group of betalains (betanin and indicaxanthin) were identified after pear cactus intake [14]. On the other hand, a study by Clifford et al. [15] indicated that using the SPE and HPLC-MS/MS methods of analysis did not allow them to find betanin in human plasma samples after both beetroot juice and whole beetroot consumption. However, the explanation for this phenomenon presented by Clifford et al. [15] is inconsistent. The authors cited suggest that before absorption, betanin is largely metabolized to unknown compounds, and therefore it is impossible to detect betalains in the plasma samples. If this had been the case, these compounds would not have been found in the urine of volunteers after the consumption of products rich in betalains. However, previous studies [16,17] indicate the presence of native betalains in the urine of volunteers after red beet intake. Therefore, other factors related to sample treatment and condition of analysis may determine the success of the analysis of betalain in plasma, as these compounds are very sensitive and may be easily degraded under the influence of different factors such as oxygen, heat, light, and pH [18]. Therefore, the analysis of betalains from plasma samples constitutes a challenging task, due to both low concentration levels and the complexity of matrices. Consequently, a successful determination of these compounds requires effective procedures of sample preparation and very sensitive equipment.

To the best of our knowledge, the Solid Phase Extraction (SPE) method with polymeric reversed phase has never been used before for sample preparation in the context of betalains content in the blood plasma. Similarly, the system of micro-high performance liquid chromatography coupled with mass spectrometry (micro-HPLC-MS/MS) has not yet been exploited as a method of analysis in the next step of a procedure. Nevertheless, there are many data on the application of the SPE method to the preparation and purification of plasma samples [19]. This method has been used for the extraction of bioactive compounds [20,21,22], elements [23], and hormones [24]. The SPE method is simple and the minimal steps involved, combined with the effectiveness of the samples purification in the analysis of trace amounts of the compounds in different materials, have caused a noticeable increase in the use of this method for samples preparation in recent years. What is more, this method enables selective extraction while avoiding the chemical changes of the test substances [25,26]. The micro-LC-MS/MS method has also been proven to be a highly effective analytical device, ensuring high sensitivity and selectivity, and when operated in the multiple reaction monitoring (MRM) mode, it is the preferred technique for quantitative analysis [27].

Taking the above into account, the aim of this study was to develop and validate a method for the determination of betalains and their metabolites in rat plasma samples based on the SPE method with the micro-HPLC-MS/MS system.

## 2. Results and Discussion

**Figure 1:**
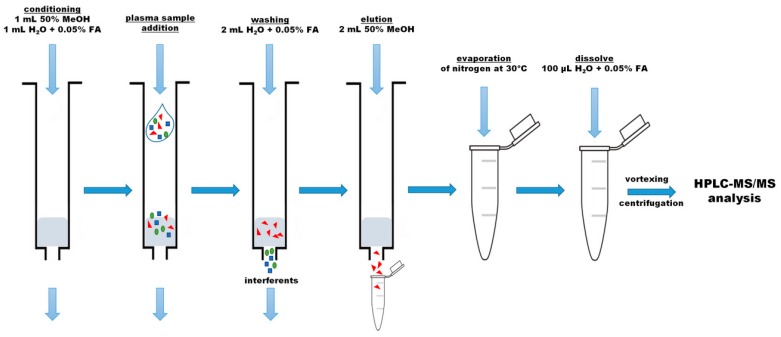
Schema of the plasma samples preparation.

Our study was focused on developing and validating a method for determination of betalains in plasma samples using combination of the SPE (with polymeric reversed phase and the mixture of methanol/water/formic acid) method with micro-HPLC-MS/MS method with elution using a solvent gradient system consisting of formic acid aqueous solution plus ammonium bicarbonate and formic acid and water acetonitrile solution plus ammonium bicarbonate. Since the individual standards of betalains are not commercially available, it was neither possible to prepare and analyze individual betalains that would be added to the blank blood plasma samples, nor to calculate the recovery ratio of the compounds. Considering the above, our data related to the recovery of betalains was determined by comparing the results obtained for the plasma fortified with the betalain preparation (1 mg betalains/L, containing 8 compounds: betanin 36.0%, isobetanin 34.8%, betanidin 5.0%, isobetanidin 2.6%, 17-decraboxy-betanin 8.4%, 17-decarboxy-isobetanina 5.4%, 2-decarboxy-neobetanina 3.1%, and neobetanin 4.6%, which were calculated as contribution of compound concentration in the total betalains concentration—the sum of the individual compounds) with the results for the betalain preparation itself (1 mg betalains/L, containing 8 compounds with identically as above the profile), using the procedure presented in Figure 1. Fermented red beet juice is characterized by a richer profile of betalain compounds than fresh red beet juice [28]; therefore, in our study this liquid was used to obtain the betalain preparation, which was used for method development. This made it possible to test the new method of betalain analysis on more compounds belonging to this very interesting and not well recognized group of phytochemicals.

In the first phase of study we tested the profile of betalain compounds in juice of fresh red beetroot, which is the primary source of these colorants in human diet as well as in the obtained betalain preparation. A fresh red beet juice was characterized by the content of five betalain compounds. Four compounds belonged to the group of betacyanins (betanin, isobetanin, 17-decarboxy-betanin, and 17-bidecarboxy-isobetanin), while the remaining one belonged to the group of betaxanthins (vulgaxanthin I). Earlier publications [29,30,31] mentioned that different red beet varieties may contain different number of compounds belonging to betalains. The differences in the number of betalains identified may result from the use in the experiment’s different varieties of plants, as well as the influence of vegetation season conditions (light, temperature, level of precipitation), climatic parameters, and cultivation conditions in which the used vegetables are grown. These observations were previously discovered also for other vegetables [32]. On the other hand, in the betalain preparation obtained from fermented red beetroot, eight betalains were identified, all belonging to the group of betacyanins. Apart from betanin, isobetanin, 17-decarboxy-betanin, and 17-bidecarboxy-isobetanin, and also betanidin, isobetanidin, neobetanin, and 2-decarboxy-neobetanin in the betalain preparation, were detected. This phenomenon might have resulted from the fact that temperature, presence of oxygen, pH value, and microorganisms’ activity may cause the conversion of some betacyanins into the decarboxylated and dehydrogenated form. For example, it was previously shown that increased temperature contributes to the conversion of betalains [33]. At the same time, vulgaxanthin I, which was found in fresh juice, was not detected in the betalain preparation. This may stem from the fact betaxanthins found in fresh red beet juice underwent a degradation process during fermentation triggered by microbial activity and conditions of the process [34].

In the second phase of study, we performed the experiment with rats, from which the plasma samples were collected after administration of both the physiological saline and the betalain preparation. The blood plasma samples obtained after stomach administration of physiological saline were used as the blank samples and the samples for betalains fortification. Next, a series of experiments was performed in order to optimize the techniques of blood plasma samples preparation. The analytical performance validation of the method applied to determine betalain compounds was determined as described earlier [35,36]. It was evaluated basing on its linearity, sensitivity, recovery, repeatability, limit of detection (LOD), and limit of quantification (LOQ). All mentioned parameters were calculated for betanin, isobetanin, betanidin, isobetanidin, neobetanin, 17-decarboxy-betanin, 17-decarboxy-isobetanin, and 2-decarboxy-neobetanin (Table 1), the compounds found in the betalains preparation. A method of least squared was applied to obtain equations of calibrations curve (*y* = a*x* + b). A good fit has been defined by the coefficient of determination (R^2^), which showed linearity when the concentrations ranged from 0.1 to 12 µmol betanin/L. The recovery values for betalain compounds detected in the enriched plasma samples ranged from 82% to 91%, with the RSD lower than 5% for all compounds analyzed. The mean recovery for all detected compounds was 86%. The highest recovery ratio was recorded for neobetanin and 17-decarboxy-betanin, while the lowest value for betanidin. Sensitivity was calculated as the calibration slope, which was appropriate. The repeatability was lower than 5% for all the compounds analyzed, and defined as the relative standard deviation (RSD) of the analytes investigated. The values of LOD and LOQ were determined by means of the signal-to-noise (S/N) ratio. The level of noise was measured basing on the chromatograms obtained for blank samples. The LOD was estimated with the 3:1 signal-to-noise ratio, while the LOQ was measured with a signal-to-noise ratio of 10:1. The LOD value for the detected betalain compounds ranged between 2.00 and 5.74 nmol/L, i.e., 2.00, 2.00, 5.74, 3.32, 4.56, 5.15, 2.26, and 5.36 nmol/L for betanin, isobetanin, betanidin, isobetanidin, neobetanin, 17-decarboxy-betanin, 17-decarboxy-isobetanin, and 2-decarboxy-neobetanin, respectively. The LOQ value for betalain compounds identified was between 6.00 and 17.22 mg/L, i.e., 6.00, 6.00, 17.22, 9.95, 13.67, 15.44, 6.77, and 16.09 nmol/L, respectively. Most importantly, the lowest values of the LOQ (6.00 nmol/L) were calculated for betanin and isobetanin, the main red beet betalains.

The method proposed in this study was characterized by a high sensitivity and excellent repeatability. Since the procedure involving the SPE method and the micro-HPLC-MS/MS analysis was used for the first time in the determination of betalains in the blood plasma, it is difficult to compare it with the data reported in the literature so far. What is more, the method presented in our study cannot be confronted with the one by Tesoriere et al. [14] (in which the extraction of plasma samples using the chloroform/methanol mixture and the HPLC-DAD analysis were performed), since this publication does not deliver any information on the validation parameters. On the other hand, literature provides data on the application of the SPE and HPLC-MS/MS methods in the analysis of other pigments (anthocyanins) occurring in plasma samples after the intake of anthocyanin-rich products [37,38]. The recovery of anthocyanin compounds in the studies cited ranged between 75–100% [37] and 65–102% [38]. The values for the LOD were between 0.003–0.7 µM [37] and 0.003–0.80 µM [38], while for the LOQ they ranged from 0.01 to 1.38 µM [37] and from 0.01 to 0.98 µM [38]. All values of betalains concentration were above the limit of quantification. Taking the above into account, the procedure developed in our study demonstrated equally satisfying validation parameters that are similar to those presented in the studies cited.

**Figure 2:**
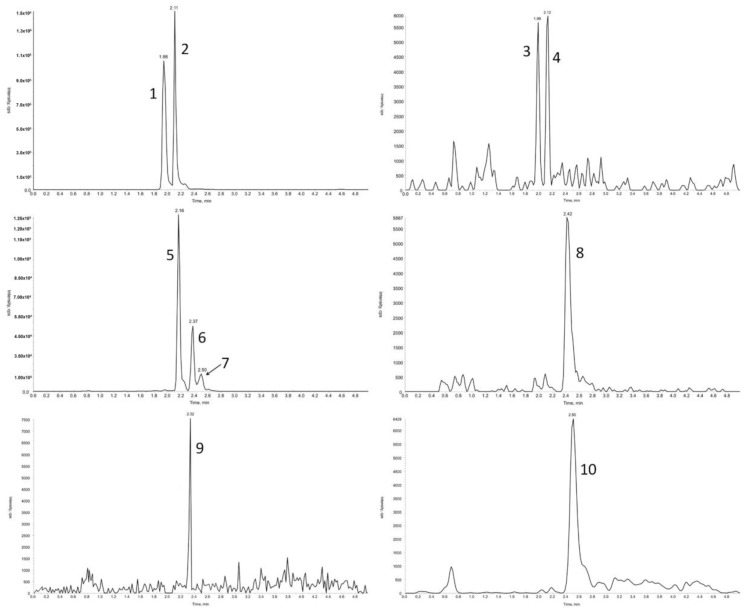
The micro-HPLC-MS/MS chromatograms of betalain compounds identified in the plasma samples (1—betanin; 2—isobetanin; 3—betanidin; 4—isobetanidin; 5—17-decarboxy-betanin; 6—17-decarboxy-isobetanin; 7—15-decarboxy-betanin; 8—neobetanin; 9—2,17-bidecarboxy-betanin; 10—2-decarboxy-neobetanin).

The study of the fate of bioactive substances consumed by humans or animals requires a comprehensive evaluation of their full profile (metabolites fingerprint) in the plasma sample, rather than only single compounds. Consequently, the method proposed by Tesoriere [14] may not be sensitive enough to determine the full profile of betalain compounds present in plasma samples, especially because it does not provide any information on the validation parameters. In our study, in the third stage, using the validated SPE method for the treatment of plasma samples and the micro-HPLC-MS/MS method for the analysis of these substances, none of betalains were detected in the plasma samples of rats (*n* = 3) treated with physiological saline. However, in the samples of rat plasma obtained after administration of the betalain preparation (50 mg betalains/kg body weight of rat), ten betalain compounds were identified (Figure 2), with eight of them being the same as in the betalain preparations (betanin, isobetanin, neobetanin, 2-decarboxy-neobetanin, 17-decarboxy-betanin and 17-bidecarboxy-isobetanin, betanidin, and isobetanidin).

Apart from native betalains present in the betalain preparation administered, two metabolites of these compounds were also identified. The two additional compounds found in the plasma of rats after red beet administration were identified by means of a comparison of their retention time, MS spectra, and the previous data [39,40,41], or through interpretation of the fragmentation spectrum obtained. Compounds with the pseudomolecular ions at *m*/*z* 507 and 463 and fragment ions at *m*/*z* 345 and 301 were identified as 15-decarboxy-betanin and 2,17-bidecarboxy-betanin, respectively. The obtained results indicated, for the first time, that native betalains are present in blood plasma after intake of products rich in betalains, as well as during processes of absorption and metabolism betalains compounds can undergo different decarboxylation processes. Wherein, the dominant compounds in the rat plasma tested were betanin (2.14 ± 0.06 µmol/L) and isobetanin (3.28 ± 0.04 µmol/L). A lower concentration was found for 17-decarboxy-betanin (0.52 ± 0.01 µmol/L), while the lowest was found for neobetanin (0.01 ± 0.00 µmol/L) (Table 2). Taking into account the results related to the profile of betalains metabolites in blood plasma, further studies are needed to explore the fate of betalains in human organism after consumption of different products rich in betalains.

## 3. Materials and Methods

### 3.1. Chemicals and Reagents

Reagents in MS grade, including methanol (MeOH), acetonitrile (MeCN), formic acid (FA), water, and ammonium bicarbonate, were purchased from Sigma Chemical Co. (St. Louis, MO, USA). 

### 3.2. Obtainment of Fresh Red Beet Juice and Betalains Preparation from Fermented Red Beet

One lot (10 kg) of red beet (*Beta vulgaris* L. subsp. *vulgaris*) was obtained from a local market in Olsztyn (Poland) and after cleaning was used to obtain the fresh red beet juice and the betalains preparation.

In laboratory conditions, a 0.42 L of fresh juice was obtained from 1 kg of red beetroots (*Beta vulgaris* L. subsp. *vulgaris*) using a juice extractor (SW-3, ZM Predom-Mesko, Skarżysko-Kamienna, Poland). After centrifugation (14,000× *g*, 20 min, 4 °C, Centrifuge 5427R, Eppendorf, Hamburg, Germany), three samples of fresh juice (1 mL each) were taken to determine the initial composition and content of betalains. Subsequently, these samples were immediately frozen and stored at −80 °C until the analysis.

The betalains preparation was obtained from the fermented red beetroot juice. Before fermentation, the remaining roots (9 kg) were chopped into ~2 mm thick strips. The obtained shredded red beetroot strips were transferred to a traditional stoneware pot and mixed with 12 L of water, 1.2% sugar, and 1.2% NaCl, and then left to start the process of spontaneous fermentation. The red beetroot pot was kept 7 days in the dark at a temperature of 24 °C. During the fermentation process, changes of pH were measured once a day using a PHM85 meter (Radiometer, Copenhagen, Denmark). The pH results obtained showed that the fermentation process was conducted properly, with a decrease in pH values ranging from 7.03 ± 0.01 to 4.06 ± 0.01. After 7 days the fermentation process was ended and, in order to obtain the betalain preparation, the fermented red beet juice was centrifuged (14,000× *g*, 20 min, 4 °C, Centrifuge 5427R, Eppendorf, Hamburg, Germany) and filtrated using a diaphragm filter (PET 0.20 µm; PPHU Q3 s.c., Bogdanka, Łódź Voivodeship, Poland). After these steps, three samples of the betalain preparation (1 mL each) were taken to determine the profile and content of betalains. Subsequently, these samples were immediately frozen and stored at −80 °C until the analysis. The remaining betalains preparation was evaporated (BÜCHI, Rotavapor R-200, Flawil, Switzerland) under nitrogen atmosphere at 30 °C and stored at −80 °C until further experiments were undertaken.

### 3.3. Animals, Administration of the Betalains Preparation, and Samples Collection

All procedures and experiments conducted complied with the guidelines in force and were approved by the Local Ethics Committee of the University of Warmia and Mazury in Olsztyn (Poland, No. 32/2015) in respect to animal testing and care of animals under study, with all possible measures having been undertaken to minimize suffering. The experiment was performed with a modified method by Passamonti et al. [42] and Talavéra et al. [43]. The study was carried out on six male *Wistar* rats, each of approximately 300 ± 10 g weight. Animals were kept in humidity and a temperature-controlled room at the Institute's animal facility, with free access to tap water. Following 24 h feed deprivation, rats were anesthetized with xylazine and ketamine, and kept alive for the time of the experiment. Next, the abdominal wall was opened and the cannulas were inserted into the stomach from the side of esophagus and duodenum. Both ends were ligated to prevent reflux. For three rats, the stomach was filled from the esophagus side with the preparation of betalains dissolved in saline to the concentration of 50 mg betalains/kg body weight of a rat. The other three rats were treated with physiological saline, following the same procedure. At 60 min after administration, blood samples were withdrawn (about 6 mL) from the *vena cava* into heparinized tubes by the lithium heparin (BD Vacutainer^®^ LH 68 I.U., BD Poland, Warsaw, Poland). The blood collected was centrifuged (500× *g*, 15 min, 1000× *g*, 10 min, 4 °C, Centrifuge MPW-351R, MPW-Med. Instrument, Warsaw, Poland), and the plasma obtained was divided depending on the analysis planned and stored at −80 °C until analyzes were undertaken.

### 3.4. Samples Preparation

The blood plasma samples obtained after stomach administration of physiological saline were used as the blank samples and the samples for betalains fortification (1 mg betalains/L). To date, there are no commercially available betalain standards that allow us to determine the individual recovery of the betalains investigated. Therefore, the usefulness of the method developed was verified on the basis of the recovery of betalains from the rat blood samples fortified with the preparation of betalain with the known concentration of these compounds (1 mg betalains/L), which was determined by the method described below (point 3.5).

Extraction of betalains from blank samples and fortified blood plasma samples was carried out with the use of the Strata^TM^-X column (Phenomenex, 33 µm, Polymeric Reversed Phase, 200 mg/3 mL, Torrance, CA, USA). The first step consisted of a two-fold dilution of plasma sample (0.5 mL) in water with 0.05% FA (0.5 mL), vortexing by 1 min and centrifugation (Centrifuge 5427R, Eppendorf, Hamburg, Germany) for 10 min (14,000× *g*, 4 °C). Then, after conditioning the Strata^TM^-X column with a mixture of methanol/water (0.5/0.5, *v*/*v*, 1 mL) and 0.05% formic acid aqueous solutions (1 mL), the diluted plasma sample was loaded. Next, the column was washed with 2 mL of water with 0.05% FA, and betalains were eluted with 2 mL of 50% MeOH. The eluent obtained was evaporated to dryness with a stream of nitrogen at 30 °C and dissolved in 100 µL of water containing 0.05% FA. Before injection into the micro-HPLC-MS/MS, the solution was centrifuged (20 min, 14,000× *g*, 4 °C). The schema of the procedure is shown in Figure 1. Each sample was prepared in triplicate.

In order to verify the method developed as to its effectiveness in detection and quantification of betalains and their metabolites, the blood plasma samples collected after into stomach administration (60 min) of the betalain preparation were examined by the procedure of extraction and purification in the same way as described above.

### 3.5. Betalains Analysis

The analysis of fresh red beet juice, the betalain preparation, the fortified blood plasma, and blood plasma was performed with the micro-HPLC-MS/MS method and HPLC-DAD method. The micro-HPLC system (LC200, Eksigent, Vaughan, ON, Canada) coupled with a mass spectrometer (QTRAP 5500, AB SCIEX, Vaughan, ON, Canada) consisting of a triple quadrupole, ion trap, and ion source of electro-spray ionization (ESI) was used to perform the analysis of betalains. The chromatographic determinations were performed on the HALO C18 column (100 mm × 0.5 mm × 2.7 µm; Eksigent, Vaughan, ON, Canada) at 45 °C with a flow rate of 25 µL/min. The elution was conducted using a solvent gradient system consisting of solvent A (0.012% formic acid aqueous solution with 5 mM ammonium bicarbonate) and solvent B (0.012% formic acid and 10% water acetonitrile solution with 5 mM ammonium bicarbonate). Gradient was as follows: 0% B (0–1.0 min), 0–20% B (1.0–2.0 min), 20–90% B (2.0–3.0 min), 90–90% B (3.0–3.8 min), 90–0% B (3.8–4.0 min), and 0% B (4.0–5.0 min). An optimal identification of compounds analyzed was achieved under the following conditions: positive ionization, curtain gas: 25 L/min, collision gas: ion-spray voltage: 5400 V, temperature: 350 °C, 1 ion source gas: 35 L/min, ion source gas: 30 L/min, declustering potential: 180 V, entrance potential: 10 V, collision energy: 40 eV, and collision cell exit potential: 27 V. The analysis of betalains λ_max_ was determined based on HPLC-DAD system (LC-20, Shimadzu, Kyoto, Japan) at 45 °C with the flow rate of 0.2 mL/min on a 150 × 2.1 mm XBridge C18 3.5 µm column (Waters, Milford, CT, USA). The elution was conducted using a solvent gradient system containing solvent A (0.012% formic acid aqueous solution with 5 mM ammonia) and solvent B (0.012% formic acid and 5% water acetonitrile solution with 5 mM ammonia). Gradient was as follows: 0–17% B (0–77 min), 17–80% B (77–80 min), 80–0% B (80–84 min), and 0% B (84–105 min). Identification of betalains was based on the comparison of their retention time, MRM (Multiple Reaction Monitoring) method with the presence of the respective and characteristic parent and daughter ion pairs (MRM ion pairs for the betalains detected are shown in Table 3, *m*/*z* values), and λ_max_ value with the previously published data [40,44]. In order to carry out quantification analysis of betalains, the previously described method of Sawicki et al. [31] was used for the preparation and quantification of external standard. Briefly, a fresh juice of red beet was 170-fold diluted and, after checking whether the preparation obtained contained only betanin and vulgaxanthin I by means of micro-HPLC-MS/MS, quantification of these compounds with a spectrophotometric method was determined according to the assay by Stintzing et al. [45]. Then, the fresh beet juice was 170-fold dissolved in Mcllvaine Buffer (pH 6.5) and reached absorbance of 0.8 ≤ A ≤ 1.0. The content of betanin and vulgaxanthin I was calculated with the following formula: BC [mg/L] = (A × DF × MW × 1000)/ε × L), where “A” stands for betanin and vulgaxanthin I absorption determined at 538 nm and 480 nm, respectively; “DF” is the dilution factor, “L” is the 1-cm path-length of the cuvette, “MW” is the molecular weight (550 g/mol for betanin and 339 g/mol for vulgaxanthin I), and “ε” is the extinction coefficients (60,000 L mol^−1^ cm^−1^ at λ = 538 nm for betanin, 48,000 L mol^−1^ cm^−1^ at λ = 480 nm for vulgaxanthin I). Quantity of betalains (betacyanins and betaxanthins) was calculated from micro-HPLC MS/MS peak area against betanin and vulgaxanthin I, respectively, as the external standards (betanin and vulgaxanthin I equivalent). The calibration curve (the range of 0.1–2 µM and 0.4–2.5 µM, respectively) was linear with a correlation coefficient of 0.997 and 0.996, respectively.

### 3.6. Statistical Analysis Method

The results are presented as mean values ± the standard error of the mean (SEM). Data were analyzed by one-way ANOVA followed by Fisher’s post-hoc test. *p* < 0.05 was considered significant. The statistical analysis was performed using Statistical Software (version 12.0; Stat Soft Corp., Tulsa, OK, USA).

## 4. Conclusions

In conclusion, this is the first study that shows elaboration of the analytical method that successfully uses the Solid Phase Extraction (with the polymeric reversed phase and the mixture of methanol/water/formic acid) and micro-high performance liquid chromatography coupled with mass spectrometry (with elution by a solvent gradient system consisting of a formic acid aqueous solution plus ammonium bicarbonate and a formic acid and water acetonitrile solution plus ammonium bicarbonate) to determine red beet betalains in rat blood plasma samples. The method proposed has considerable potential in assessing the fate of betalains—strong bioactive compounds—in the human body after consumption of a number of food products containing these natural colorants. This may, in turn, significantly contribute to recognizing the beneficial role of betalains for human health. What is more, this method gives options for quantifying small molecule analytes with low and very low concentrations.

## Figures and Tables

**Table 1:** Data of betalains quantification.

Betalains Compounds	R^2^	LOD (nmol/L)	LOQ (nmol/L)	Recovery (%)	RSD (%)
Betanin	0.999	2.00	6.00	84	2.3
Isobetanin	0.999	2.00	6.00	83	2.8
Betanidin	0.999	5.74	17.22	82	1.8
Isobetanidin	0.999	3.32	9.95	89	3.7
Neobetanin	0.999	4.56	13.67	91	4.7
17-Decarboxy-betanin	0.999	5.15	15.44	91	4.9
17-Decarboxy-isobetanin	0.999	2.26	6.77	86	2.0
2-Decarboxy-neobetanin	0.999	5.36	16.09	83	1.3

**Abbreviations:** R^2^—coefficients of determination; LOD—limit of detection; LOQ—limit of quantification; RSD—related standard deviation.

**Table 2:** Content of betalains in rat plasma samples (µmol/L).

Compounds	Content
**Native**	
betanin	2.14 ± 0.06 ^b^
isobetanin	3.28 ± 0.04 ^a^
betanidin	0.02 ± 0.00 ^f^
isobetanidin	0.02 ± 0.00 ^f^
neobetanin	0.01 ± 0.00 ^f^
17-decarboxy-betanin	0.52 ± 0.01 ^c^
17-decarboxy-isobetanin	0.13 ± 0.01 ^d^
2-decarboxy-neobetanin	0.02 ± 0.00 ^f^
**metabolites**	
15-decarboxy-betanin	0.08 ± 0.01 ^e^
2,17-bidecarboxy-betanin	0.04 ± 0.00 ^f^

Data are expressed as mean ± SEM (*n* = 3). Means followed by the different letters are significantly different (*p* < 0.05).

**Table 3:** Betalains identified in fresh juice, preparation, and plasma.

Compounds	R_t_ (min)	λ_max_ (nm)	MRM Ion Pairs	Sample
**Betaxanthins**				
glutamine-betaxanthin (vulgaxanthin I)	1.00	475	340/323	fresh juice
**Betacyanins and Their Derivatives**				
betanin	1.98	537	551/389	fresh juice, preparation, plasma
betanidin	1.99	539	389/345	preparation, plasma
isobetanin	2.11	537	551/389	fresh juice, preparation, plasma
isobetanidin	2.12	539	389/345	preparation, plasma
17-decarboxy-betanin	2.16	507	507/345	fresh juice, preparation, plasma
2,17-bidecarboxy-betanin	2.32	-	463/301	plasma
17-decarboxy-isobetanin	2.37	505	507/345	fresh juice, preparation, plasma
neobetanin	2.42	471	549/387	preparation, plasma
15-decarboxy-betanin	2.50	-	507/345	plasma
2-decarboxy-neobetanin	2.50	485	505/343	preparation, plasma

**Abbreviations:** R_t_—retention time; MRM—multiple reaction monitoring.
